# Synthesis and Characterization of Zinc and Vanadium Co-Substituted CoFe_2_O_4_ Nanoparticles Synthesized by Using the Sol-Gel Auto-Combustion Method

**DOI:** 10.3390/nano12050752

**Published:** 2022-02-23

**Authors:** Parvin Imanipour, Saeed Hasani, Amir Seifoddini, Marcin Nabiałek

**Affiliations:** 1 Department of Mining and Metallurgical Engineering, Yazd University, Yazd 89195-741, Iran; elmira.ip7070@gmail.com (P.I.); seifoddini@yazd.ac.ir (A.S.); 2 Department of Physics, Częstochowa University of Technology, 42-200 Częstochowa, Poland; marcin.nabialek@pcz.pl

**Keywords:** cobalt ferrite, doping elements, magnetic properties, coercivity, sol-gel method

## Abstract

In recent years, cobalt ferrite has attracted considerable attention due to its unique physical properties. The present study aimed to produce cobalt ferrite magnetic nanoparticles doped with zinc and vanadium using the sol-gel auto-combustion method. For this purpose, Co_1−x_Zn_x_Fe_2−y_V_y_O_4_ (where x = 0.0, 0.1, 0.2, 0.5 and y = 0.00, 0.05, 0.15, 0.25) precursors were calcined at 800 °C for 3 h. The prepared samples were characterized with the X-ray diffraction (XRD) method in combination with Rietveld structure refinement, field emission scanning electron microscopy (FE-SEM), Fourier transform infrared spectroscopy (FT-IR), and vibrating sample magnetometery (VSM). The XRD patterns confirmed the formation of crystalline spinel structure for all samples. However, the diffraction peaks of hematite and iron vanadium oxide phases were observed in the patterns of some doped samples. The average crystallite size for all the synthesized samples was found to be in the range of ~45–24 nm, implying that it decreased by simultaneously doping cobalt ferrite with Zn and V. The FT-IR spectrum confirmed the formation of the spinal structure of ferrite through the observed vibrational bands assigned to the tetrahedral (υ_2_) and octahedral (υ_1_) interstitial complexes in the spinel structure. The FE-SEM images showed that morphology, average grain size, and agglomeration of the synthesized powders were affected by doping, which was due to the interactions of the magnetic surface of nanoparticles. The VSM curves demonstrated that saturation magnetization and coercivity values changed in the range of 30–83 emu/g and from 27–913 Oe, respectively. These changes occurred due to the alteration in cation distribution in the spinel structure. This can be attributed to the change in superexchange interactions between magnetic ions by co-substitution of Zn and V ions in Cobalt ferrite and the changes in magnetocrystalline anisotropy.

## 1. Introduction

In recent decades, the synthesis of nanostructured spinel ferrites with the general formula MFe_2_O_4_ (where M = Co, Ni, Mg, Mn, etc.) has been investigated in order to improve their unique properties such as magneto-optical properties [1,2,3], catalytic properties [4,5], electrical resistivity [6], magneto-resistivity [7], biocompatibility [8,9,10], porosity [11], etc. The spinel ferrites have a cubic unit cell containing 56 atoms, where 32 of them are oxygen anions, and the other 24 atoms are metallic cations of which only eight occupy tetrahedral sites (A-site) while the other 16 atoms occupy octahedral sites (B-site) [12,13]. Among spinel ferrite nanoparticles, pure cobalt ferrite and its substituted compounds have attracted the considerable attention of numerous researchers in recent decades due to the reliable saturation magnetization (*M_s_*) [14], moderate magnetocrystalline anisotropy (*K*) [15,16], high coercivity (*H_c_*) [17], high Curie temperature (*T_c_*) [18], and thermal stability [19,20]. It is accepted that the chemical, electrical, and magnetic properties of cobalt ferrite nanoparticles are dependent on the chemical composition, cation distribution in the two sub-lattices of A- and B-sites, morphology, surface properties, and preparation method [21,22,23,24]. For instance, these nanoparticles are often synthesized by various routes such as co-precipitation [25,26], ultrasound irradiation, solution combustion [27], solid-state reaction [28], the conventional ceramic method [29], ball milling [30], and sol-gel auto combustion [31] methods. Among these methods, the sol-gel method is known as a suitable technique to produce nanoparticles with high homogeneity, proper microstructure, and other suitable properties [32,33,34].

On the other hand, cation distribution in the nanoparticles controls their magnetic behavior, so that these nanoparticles can show a ferromagnetic, super-paramagnetic, or antiferromagnetic behavior [6,35,36,37]. Therefore, the addition or doping of other elements instead of the Fe- and/or Co- sites has been the subject of much research [38,39,40,41]. It is reported that Zn [42,43,44] and V [43,45] can strongly affect the structural and physical properties of cobalt ferrite. Substitution of Zn^2+^ ion on the Fe- or/and Co- site in cobalt ferrite considerably was reported [27,42,46,47]. For instance, Kumar et al. [41] studied the substitution of Zn^2+^ at the Fe site of CoZn_x_Fe_2−x_O_4_ nanoparticles and found that the saturation magnetization and magnetocrystalline anisotropy are increased for x ≥ 0.15, but the coercivity is decreased by adding Zn^2+^. On the other hand, the effect of vanadium doping on the magnetic properties of cobalt ferrite has been investigated [43,45]. The obtained results showed that the V-doped cobalt ferrite indicates a higher coercivity than the pure cobalt ferrite. Therefore, Zn and V, as two doping elements, have the opposite effect on the magnetic properties of these nanoparticles. In this regard, adding more than one element is known as one of the most effective methods to modify the structural and magnetic properties of these nanoparticles [48,49,50]. Therefore, in the present study, co-substitution of Zn and V at Co and Fe sites, respectively, and their effects on the structural and magnetic properties of CoFe_2_O_4_ nanoparticles were investigated.

## 2. Materials and Methods

### 2.1. Materials

Merck Company supplied all of the materials used in this work with analytic grade reagents. The chemical reagents were Cobalt (ΙΙ) nitrate hexahydrate (Co(NO_3_)_2_·6H_2_O, ≥99%), iron (ΙΙΙ) nitrate nonahydrate (Fe(NO_3_)_3_·9H_2_O, ≥99%), Zinc nitrate tetrahydrate (Zn(NO_3_)_2_·4H_2_O, ≥99%), ammonium monovanadate (NH_4_VO_3_, ≥99%), citric acid (≥99.5%), and ammonia solution (≥25%). Additionally, double-distilled water (DDW) was used as the solvent.

### 2.2. Samples Preparation

**Figure 1:**
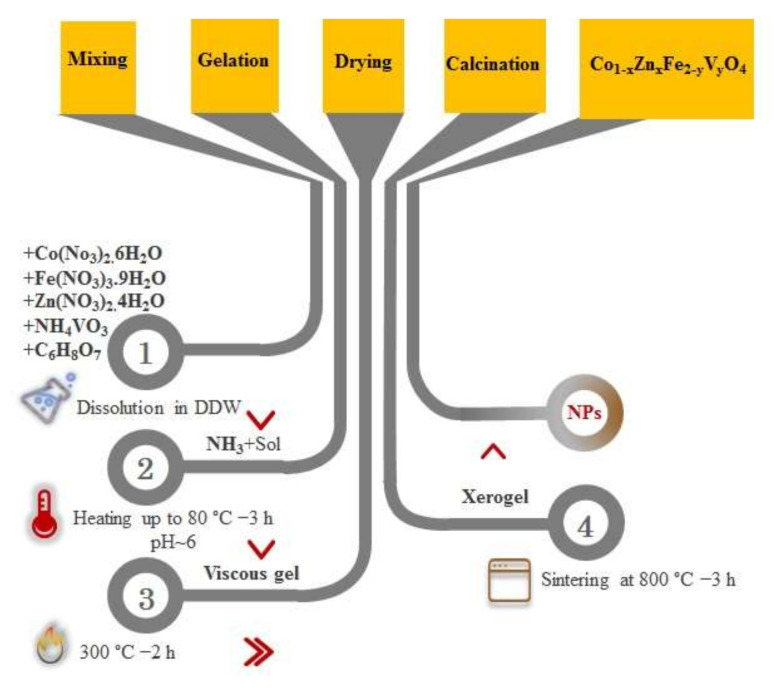
A schematic diagram representing the synthesis process used in the present study.

Co_1−x_Zn_x_Fe_2−y_V_y_O_4_ (where x = 0.0, 0.1, 0.2, 0.5 and y = 0.00, 0.05, 0.15, 0.25) nano-powders were synthesized via the sol-gel auto combustion method. The chemical composition of these specimens is presented in Table 1. The stoichiometric amounts of nitrate precursors along with citric acid were weighted as 1:2 ratios and were dissolved in DDW using a magnetic stirrer. Then, ammonia solution is added dropwise to maintain the pH at ~6. By adding a small amount of ammonia solution, a slight increase in the turbidity of the solution was observed. After that, the precursor’s mixture was continuously stirred at 80 °C for 3 h until the puffy dark brown gel appeared. With an increase in temperature up to 300 °C, the viscous gel began to froth, and a dried gel achieves. Then, a brownish-black powder was observed. Finally, the prepared powder was sintered at 800 °C for 3 h to obtain crystalline cobalt ferrite nanoparticles. To further clarify the production process, a schematic diagram representing the synthesis process is shown in Figure 1.

### 2.3. Characterization Technique

Determination of phase purity and their identification was done with the X-ray diffraction (XRD) method using a Philips powder diffractometer PW1730 with Cu kα radiation. All of the samples were tested in continuous mode from 20 to 70°. The constitutional analysis in a more accurate profile of all the samples was studied by Rietveld refinements that was performed using PANalytical X’Pert HighScore Plus software (version: 3.0e (3.0.5)). The size and morphology of the samples were analyzed using field emission scanning electron microscopy (FE-SEM; MIRA 3 Tescan, Kohoutovice, Czech Republic.). The thermal behavior of the xerogels was investigated by the non-isothermal differential thermal analysis-thermogravimetry (DTA-TG; Bahr 503, Hüllhorst, Germany), at a heating rate of 20 °C/min up to 1200 °C under air atmosphere. The Fourier transformed infrared (FT-IR; Avatar, Thermo, Waltham, USA) spectra of the samples A, F, and K were recorded in the range 400–4000 cm^−1^. Moreover, magnetization measurements were carried out using a vibration sample magnetometery (VSM; Magnetic Daghigh Daneshpajouh, Kashan, Iran) at room temperature.

## 3. Results

### 3.1. Thermal Behavior

**Figure 2:**
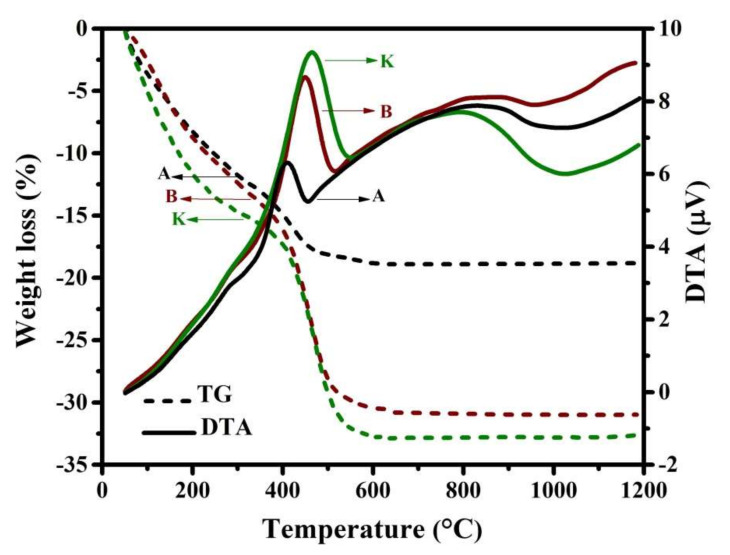
DTA-TG curves related to the samples A, B, and K at a heating rate of 20 °C/min.

The thermal behavior of the xerogels is investigated using the non-isothermal DTA-TG analyses. For instance, the DTA-TG curves of xerogels related to the samples A, B, and K are presented in Figure 2. This figure illustrates the evolution of TG (shown as dashed lines) and DTA curves. It is obvious that the evaporation of water molecules, decomposition process, and formation of spinel ferrite could be completed below 800 °C, which is in agreement with reported by literature [43,47]. Additionally, the DTA curves show obviously three exothermic peaks in the range of (i) ~RT–292, (ii) ~310–552, and (iii) ~556–1000 °C. The temperature characteristics of these peaks, along with the weight loss percentage, are presented in Table 2. It is notable that the first and second DTA peaks overlap each other, but are well separated from the third DTA peak. The first exothermic peak is mainly due to the combustion of some volatiles. Of course, evaporation of the structural water also occurs in this temperature range, which can be effective for the weight loss observed in this stage. However, the result of these reactions occurring in this temperature range is observed, as an exothermic step.

The second exothermic peak and corresponding TG profile are related to the decomposition of the nitrate salts and other organic compounds, as well as the formation of the spinel ferrite. However, it can be observed that this exothermic peak becomes higher and broader with co-substitution of Zn and V at Co and Fe sites, respectively, which can be due to an increase in the formation enthalpy. In addition, the weight loss at this stage increases by doping.

Moreover, the third exothermic DTA peak (as a broad peak) referred to the powder densification, as mentioned in the literature [47], and therefore no noticeable weight loss is observed at this stage.

### 3.2. X-ray Diffraction and Structural Studies

**Figure 3:**
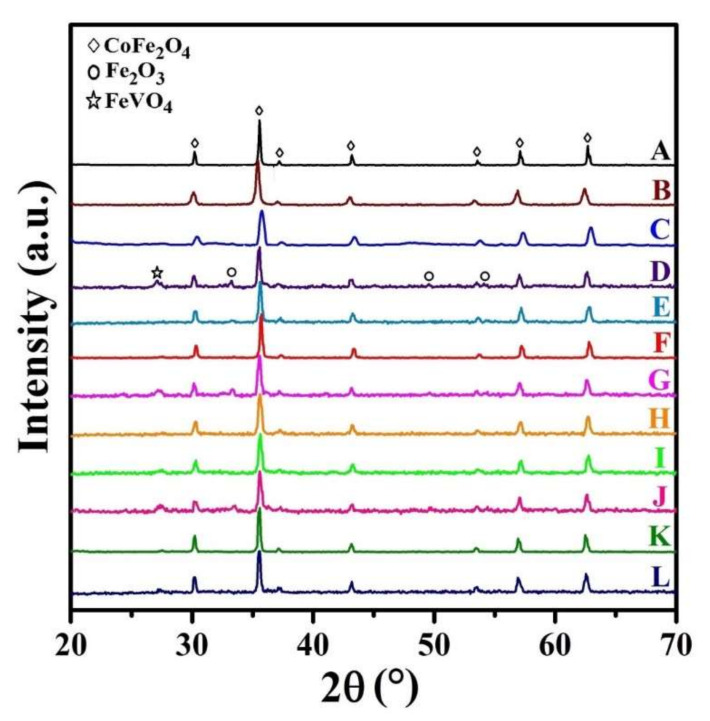
XRD patterns of the synthesized samples.

The XRD patterns of all samples are presented in Figure 3. As seen, there are several typical diffraction characteristic peaks related to the cubic spinel structures, which is assigned to (220), (311), (222), (400), (422), (511), and (440) planes (JCPDS reference card #96-591-0064). However, by co-substitution of Zn and V at Co and Fe sites, respectively, the diffraction peaks of hematite (Fe_2_O_3_, JCPDS reference card #96-591-0083) and iron vanadium oxide (FeVO_4_, JCPDS reference card #00-030-0667) phases appear in the XRD patterns of some samples.

In our previous study [43], it was reported that the amount of the impurity/secondary phases is sharply increased by co-substitution V atoms at Co-sites, while the impurity/secondary phases of Fe_2_O_3_ and FeVO_4_ are only observed in some of the XRD patterns, where V atoms occupy Fe-sites (as presented in Figure 3). Therefore, it could be concluded that vanadium atoms are easier to occupy iron positions. Thus, the substitution of vanadium for iron will induce the replacement of Fe^3+^ by V^3+^ cations, and therefore the pure spinel structure of cobalt ferrite can be observed (sample C), which is in very good agreement with the observations by Heiba et al. [45].

It is notable that the cation distribution is changed with the presence of doping elements. Consequently, the cation distribution may be changed in the doped samples. Although the vanadium ions mainly occupy the tetrahedral sites, some octahedral sites may be occupied by adding a low amount of vanadium [45], which is in good agreement with that obtained in XRD patterns of samples D, G, and J. The presence of a secondary phase in these samples can be due to this phenomenon.

The average crystalline size of cobalt ferrite nanoparticles are calculated from line width of (311) peak using Scherer’s formula after taking into account the instrumental broadening [51];

(1) $$D=\frac{0.89\lambda }{\beta \mathrm{Cos}\theta }$$

where ‘*D*’ is the crystallite size; ‘*λ*’ is the X-ray wavelength (1.5406 Å); ‘*β*’ is the full width at half maximum (FWHM) for (311) peak, and ‘*θ*’ is the diffraction angle [52]. The obtained results are presented in Table 3. As seen, the average crystallite size in the doped samples is smaller. However, the average crystallites size decreases initially with the doping of zinc but increases again with the doping by more significant amounts of zinc, which is in good agreement with the other observation [46]. The result specifies that the lower amount of zinc (x = 0.0, 0.1, and 0.2) delays the growth of the crystallite size; meanwhile, the larger amount of zinc favors the growth of the crystallite size at the nucleation centers, which resulted in higher crystallite size.

In addition, the structural parameters for all synthesized samples were calculated using the Rietveld refinement method. These parameters are listed in Table 3. As seen, there is a good agreement between the results obtained by Scherer’s formula and Rietveld method. However, there is a slight difference between them, which can be due to the presence of hematite impurities in some samples, because one of the hematite peaks overlaps with the (311) peak around 36°.

On the other hand, the values of the lattice constant, the unit cell volume, and the X-ray density of Co_1−x_Zn_x_Fe_2−y_V_y_O_4_ nanoparticles are determined using the equations given in Ref. [43]. These results are also presented in Table 3. As seen, the doping by Zn increases the values of the lattice constant/volume, because the Zn^2+^ ions (0.82 Å) are larger than the Co^2+^ ions (0.75 Å) [47]. Additionally, the crystallographic theoretical density values increase with an increase in zinc and vanadium content up to 0.2 and 0.15, respectively. The crystallographic density is dependent on the molecular weight of atoms and the lattice constant, which can be affected by doping.

As presented in Table 3, the lattice parameter is decreased by an increase in the vanadium value. This could be correlated with the variation of the cation distribution. On the other hand, the addition of zinc and vanadium ions can be changed the cation distribution out of the ideal state, so cobalt cations have more opportunity to occupy the tetrahedral sites. Hence, Fe^3+^ ions located in the tetrahedral sites are forced to leave their location and shift to the octahedral sites. However, according to the ionic radius arrangement of these elements (V^5+^ (0.36 Å) < Fe^3+^ (0.64 Å) < Co^2+^ (0.75 Å) < Zn^2+^ (0.82 Å)), with the substitution of zinc instead of cobalt, it is expected to increase the lattice parameter. Furthermore, substitution of elements will cause the structure strain and prevent the growth of crystallites.

### 3.3. Microstructural Observations

**Figure 4:**
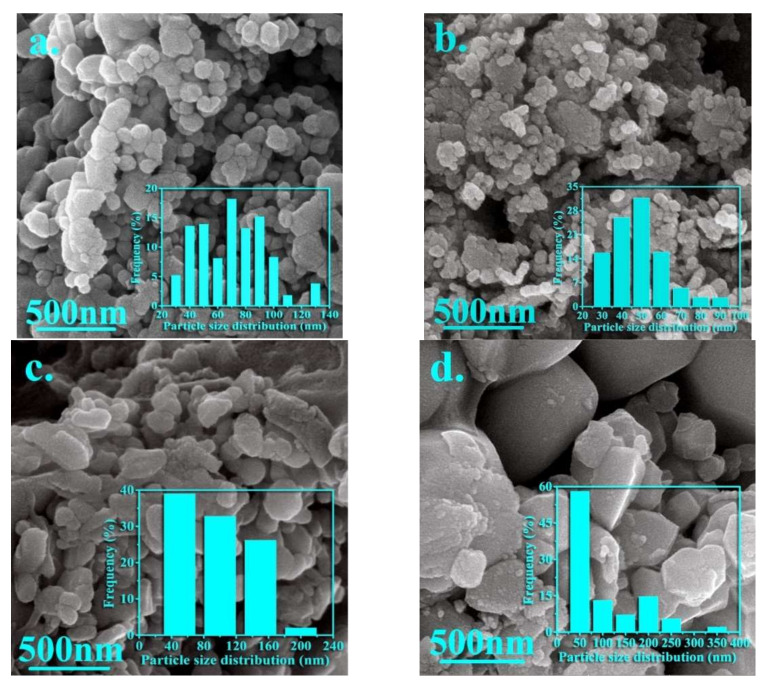
FE-SEM images of the nanoparticles synthesized in the samples (**a**) A, (**b**) B, (**c**) J, and (**d**) K.

Figure 4 shows the morphologies along with particle size distribution in the synthesized nanoparticles for samples A, B, J, and K. Additionally, the average grain size for these samples, extracted from FE-SEM images, is listed in Table 3.

It is notable that an inhomogeneous strain distribution can be created due to the difference in ionic radii of the cations, so that inhomogeneous strain distribution can form the particles with a random size and irregular shape. As seen in Figure 4a, the uniform nanoparticles with refined grains, spherical shapes, and some agglomeration can be observed for sample A. Figure 4b shows the spherical and flaky shape for the nanoparticles synthesized in sample B. Additionally, it can be seen that agglomeration is increased by adding zinc, but the average grain size is decreased from 63 to 46 nm. However, two types of particles with different morphologies can be observed by simultaneously adding zinc and vanadium, so that larger particles can be related to the presence of impurity particles (hematite and iron vanadium oxide). However, it can be seen that the agglomeration is severely reduced by simultaneously adding elements.

The optimal ratio of both elements results in a homogeneous distribution of particles. As observed in these pictures, the particles are mostly spherical or semi-spherical. However, it is notable that the agglomeration of nanoparticles can be due to the interactions of the magnetic surface of nanoparticles. Thus, the morphology, uniformity, and agglomeration of synthesized nanoparticles can be changed in the presence of doping elements (please see Figure 4c,d).

It is notable that the average crystallite/particle sizes calculated by XRD and FE-SEM respectively are different from each other, which can be due to the formation of every particle by aggregation of a large number of crystallites or grains and also strain over the surface of the nanoparticles can cause on the further broadening of XRD peak profiles.

### 3.4. FTIR

**Figure 5:**
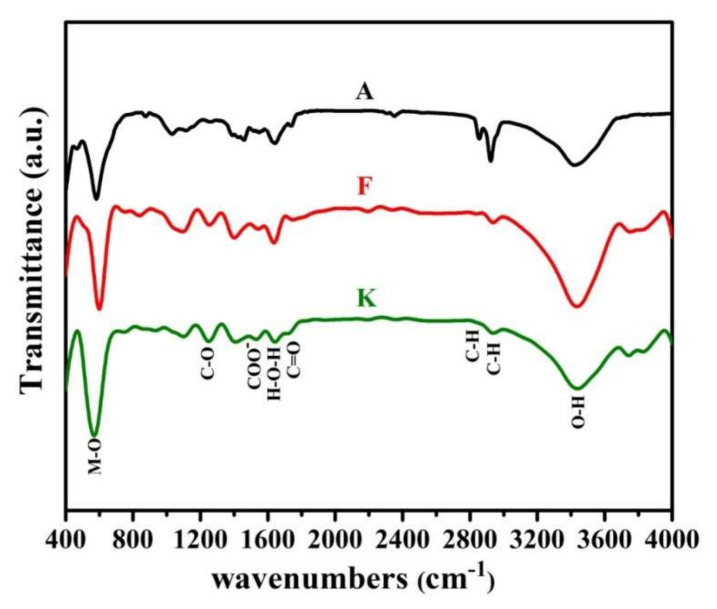
FT-IR spectra for the synthesized samples A, F, and K.

FTIR Spectroscopy is a very useful tool to investigate the vibrational properties of molecules in the synthesized samples. For instance, the FTIR spectra related to the nanoparticles synthesized in the samples A, F, and K are presented in Figure 5. The results indicate that products of all three samples A, F, and K have the same molecular structure. As seen, two main absorption bands (M-O) at 489 cm^−1^ and 593 cm^−1^ are observed, which are the characteristic of spinel ferrite [53]. The first and the second bands of absorption are related to intrinsic lattice vibrations of the octahedral (*v*_1_) and tetrahedral (*v*_2_) sites. The difference in vibrational frequencies is due to the long bond length of oxygen-metal ions in the tetrahedral positions, which is dependent upon the bonding force, cation mass, and oxygen-metal distance [54,55]. In addition, it is observed that co-substitution of Zn and V ions causes at first a slightly shift of band positions toward the high wave number and then to the low wavenumber. As mentioned, it can be due to the change in the crystalline size of nanoparticles and also the cation distribution between the tetrahedral and octahedral sites. The higher wavenumber shows the vibration of Fe^3+^–O^2−^ at tetrahedral sites and the lower wavenumber depicts the trivalent metal-oxygen vibration at octahedral sites.

In addition, other peaks are marked, and sufficient description of them has been provided in previous articles [17,56].

### 3.5. Magnetic Properties

**Figure 6:**
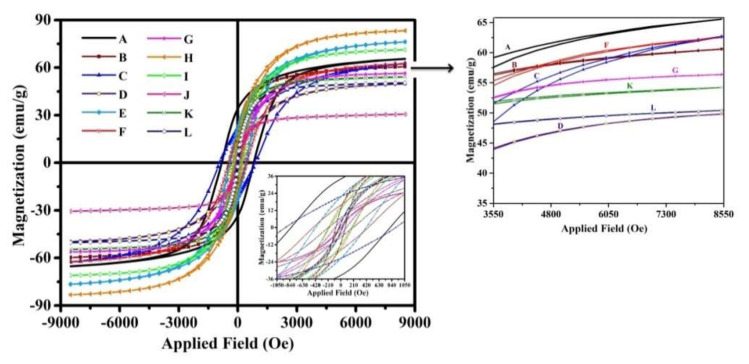
M-H loops of all synthesized nanoparticles at room temperature.

The hysteresis loops (*M*–*H*) of Co_1−x_Zn_x_Fe_2−y_V_y_O_4_ nanoparticle with (x = 0.0, 0.1, 0.2, 0.5 and y = 0.00, 0.05, 0.15, 0.25) were laid out in Figure 6. These magnetic measurements provide information about magnetic parameters such as saturation magnetization (*M_s_*), coercivity (*H_c_*), remanent magnetization (*M_r_*), magnetic anisotropy (*K*), magnetic moment (*n_B_* in Bohr magneton), and squareness ratio (*M_r_/M_s_*) ratio that is listed in Table 4. It is necessary to mention that the values of *K*, *n_B_*, and *M_r_/M_s_* ratio were calculated by the equations presented in previous articles [43,47].

**Figure 7:**
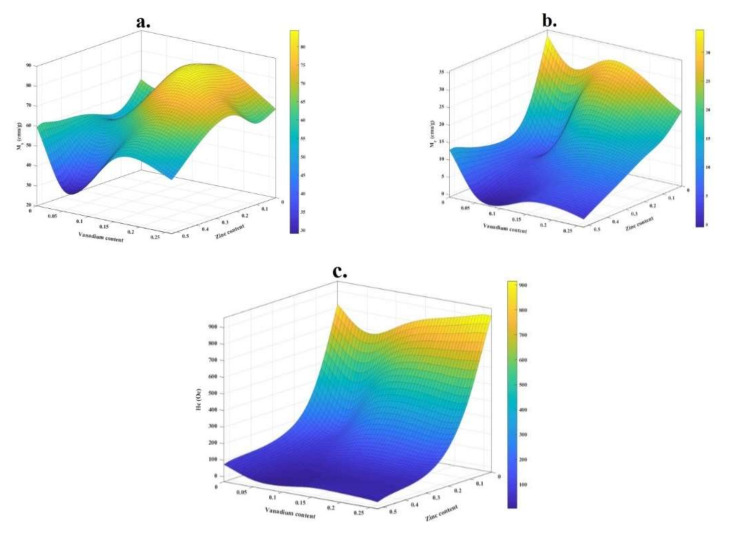
Variation of (**a**) *M_s_*, (**b**) *M_r_*, and (**c**) *H_c_* as a function of Zn and V contents.

For better comparison, the values of *M_s_*, *M_r_*, and *H_c_* versus dopants contents are shown in Figure 7a–c, respectively.

As presented, sample H represented the highest *M_s_*, (Figure 7a) so that the saturation magnetization in this sample is ~27% higher than in sample A, which indicates that adding intermediate amounts of doping elements can have the more significant effect on this magnetic property. It can be explained based on cation distribution and the exchange interactions between A and B sites. Additionally, it is reported that this magnetic property can be increased with increasing vanadium content due to spin canting and disorder in the surface spin [57]. Of course, the effect of the presence of impurity phases on the saturation magnetization of the samples is also noticeable, so that, as expected, the presence of impurity phases reduces this property.

On the other hand, it is shown that the addition of Zn decreases *M_r_* in the synthesized nanoparticles (Figure 7b), so that the minimum of *M_r_* is observed for sample J (~0.94 emu/g). It is also well observed that sample J has the lowest amount of coercivity, which is in good agreement with that reported in the literature [47,58]. However, adding vanadium prevents this reduction.

The highest value of coercivity is obtained for sample C, which is equal to ~913 Oe (Table 4). Figure 7c shows that with the addition of vanadium, this magnetic parameter increases, while with the addition of zinc, it decreases. This magnetic parameter is affected by the presence of impurity phases and various structural defects, i.e., dislocations, grain boundaries, anisotropy, and precipitates.

As seen in Table 4, the amount of *M_r_* has a good relationship with the amount of *K* in which with adding zinc, the value of *K* decreases to ~0.04 erg/g. The maximum value of *K* is obtained for sample A, which can be due to the presence of Co^2+^ ions in octahedral sites. Furthermore, with the co-substitution of zinc and vanadium, the presence of cobalt ions in octahedral sites is reduced due to the changes in cation distribution.

Finally, it can be concluded that the magnetic behavior of the samples can be affected by three important factors; (i) the presence of secondary/impurity phases, (ii) cation distribution in the spinel structure, and (iii) the size of the crystallites/grains.

## 4. Conclusions

The effect of zinc and vanadium doping on the microstructure, and structural and magnetic properties of cobalt ferrite synthesized by modified sol-gel method was investigated. The formation of cubic spinel structure is confirmed by XRD. The cation distribution also showed that V^5+^ tended to be located in tetrahedral sites. Nevertheless, by adding a low concentration of these elements, it can also occupy octahedral sites. Hence, the formation of a secondary/impurity phase such as hematite and vanadium iron oxide in these conditions is inevitable. The ionic radius arrangement of these elements (V^5+^ (0.36 Å) < Fe^3+^ (0.64 Å) < Co^2+^ (0.75 Å) < Zn^2+^ (0.82 Å)), it makes sense to create and increase stress and strain in the spinel lattice, which can inhibit the growth of crystals. FTIR spectra revealed the absorption peaks near 489 and 593 cm^−1^ that affirms the ferrite behavior of the samples. The FE-SEM micrographs revealed some fine-sized agglomerated semi-spherical-shaped particles. The agglomerated grains indicated the strong interactions between the particles and continuity of the grain growth during sintering. The hysteresis curves showed that the increase and decrease in magnetic properties could be due to the replacement cations.

## Figures and Tables

**Table 1:** Chemical composition of the synthesized samples.

Sample Code		Chemical Composition
A		CoFe_2_O_4_
B		Co_0.5_Zn_0.5_Fe_2_O_4_
C		CoFe_1.75_V_0.25_O_4_
D		Co_0.9_Zn_0.1_Fe_1.95_V_0.05_O_4_
E		Co_0.9_Zn_0.1_Fe_1.90_V_0.15_O_4_
F		Co_0.9_Zn_0.1_Fe_1.75_V_0.25_O_4_
G		Co_0.8_Zn_0.2_Fe_1.95_V_0.05_O_4_
H		Co_0.8_Zn_0.2_Fe_1.85_V_0.15_O_4_
I		Co_0.8_Zn_0.2_Fe_1.75_V_0.25_O_4_
J		Co_0.5_Zn_0.5_Fe_1.95_V_0.05_O_4_
K		Co_0.5_Zn_0.5_Fe_1.85_V_0.15_O_4_
L		Co_0.5_Zn_0.5_Fe_1.75_V_0.25_O_4_

**Table 2:** The DTA peak temperatures and mass loss occurred in every stage, extracted from DTA-TG curves (presented in Figure 2).

Samples		DTA Peak Temp. (°C)						TG-Mass Loss (%)		
		i		ii		iii		i		ii
A		277.8		409.8		826.2		13.1		17.6
B		282.0		450.1		852.5		15.6		28.8
K		292.0		463.9		792.6		16.4		31.6

**Table 3:** Structural parameters of the synthesized samples.

Sample Code		Crystallite/Grain Size (nm)						X-ray Density (gr/cm^3^)		Lattice Parameter (Å)				Volume (Å^3^)
		XRD				FE-SEM								
		Debye-Scherrer’ Formula		Rietveld Analysis						Rietveld Analysis		XRD		
A		45		74.3		63.9		5.32		8.39275		8.364		585.11
B		30.33		31.1		46.6		5.23		8.42654		8.409		594.61
C		32.39		34.3		-		5.41		8.38041		8.318		575.53
D		29.11		46.9		-		5.27		8.38874		8.387		590.11
E		30.98		42.4		-		5.33		8.38933		8.357		583.83
F		36.44		52.0		-		5.36		8.38883		8.345		581.06
G		23.86		43.1		-		5.32		8.39411		8.364		585.22
H		25.54		36.9		-		5.73		8.40177		8.371		586.62
I		24.67		39.1		-		5.32		8.40215		8.364		585.22
J		32.35		39.5		65.31		5.31		8.42509		8.367		585.92
K		38.31		61.0		59.3		5.29		8.42363		8.377		588.01
L		32.35		49.5		-		5.30		8.42048		8.374		587.31

**Table 4:** Magnetic properties of the synthesized nanoparticles.

Sample Code	*M_s_* (emu/g)	*H_c_* (Oe)	*M_r_* (emu/g)	*K* × 10^4^ (erg/g)	*n_B_*	*M_r_/M_s_*
A	65.6	820.3	33.9	5.50	2.75	0.51
B	60.7	84.4	13	5.22	2.54	0.21
C	62.7	913.6	21.8	5.84	2.63	0.34
D	49.8	204.9	6.8	1.04	2.09	0.13
E	76.4	415.9	21.7	3.24	3.20	0.28
F	62.6	458	15.8	2.92	2.62	0.25
G	56.3	96.8	5	0.55	2.36	0.08
H	83.1	202.6	15	1.71	3.49	0.18
I	71.2	184.3	12.8	1.33	2.99	0.17
J	30.5	15.7	0.94	0.04	1.28	0.03
K	54.3	35.8	3.5	0.19	2.28	0.06
L	50.7	27.2	2.9	0.14	2.12	0.05
